# Clinical Evaluation of the Optical Filter for Autofluorescence Glasses for Oral Cancer Curing Light Exposed (GOCCLES^®^) in the Management of Potentially Premalignant Disorders: A Retrospective Study

**DOI:** 10.3390/ijerph19095579

**Published:** 2022-05-04

**Authors:** Carlo Lajolo, Mariateresa Tranfa, Romeo Patini, Antonino Fiorino, Teresa Musarra, Roberto Boniello, Alessandro Moro

**Affiliations:** 1 Department of Head, Neck and Sense Organs, Università Cattolica del Sacro Cuore, Fondazione Policlinico Universitario A. Gemelli IRCCS, 00168 Rome, Italy; carlo.lajolo@unicatt.it (C.L.); mariateresa.tranfa01@icatt.it (M.T.); fiorinodr.antonino@gmail.com (A.F.); roberto.boniello@policlinicogemelli.it (R.B.); alessandro.moro@unicatt.it (A.M.); 2 Department of Women’s, Children’s and Public Health Sciences, Fondazione Policlinico Universitario A. Gemelli IRCCS, 00168 Rome, Italy; teresa.musarra@guest.policlinicogemelli.it

**Keywords:** autofluorescence, toluidine blue, dysplasia, oral potentially malignant disorders, oral cancer, biopsy, retrospective study

## Abstract

Background: Any oral potentially malignant disorders (OPMDs) must be regularly monitored through clinical examination to detect any possible malignant transformation. Conventional intraoral exams, however, can be difficult because these conditions may resemble benign lesions. For this reason, several non-invasive diagnostic technologies have been developed to help the clinician in detecting and distinguishing between cancerous and benign lesions. Epithelial dysplasia can be considered the most important predictor of malignant evolution. Therefore, in this study we aim to evaluate the ability of an optical filter for autofluorescence Glasses for Oral Cancer Curing Light Exposed (GOCCLES^®^) and of toluidine blue staining in identifying dysplastic areas in patients with OPMDs. Methods: In this retrospective study, medical records, photographs and videos of 25 patients with oral lesions were analyzed. Forty-two biopsy samples in 25 patients with OPMDs and at least one suspicious oral mucosa lesion that were evaluated in white light, autofluorescence with optical filter GOCCLES^®^, toluidine blue staining and then biopsied with histopathological analysis were analyzed. Results: The sensitivity and specificity for the autofluorescence evaluation with GOCCLES^®^ for identifying dysplasia or carcinoma were 66% and 48%, respectively. The positive and negative predictive values were 34% and 77%, respectively, and the accuracy was 53%. The sensitivity and specificity for toluidine blue staining were 91% and 68%, respectively. The positive and negative predictive values were 55% and 95%, respectively, and the accuracy was 75%. Conclusions: The optical filter for autofluorescence (GOCCLES^®^) and toluidine blue staining are simple, inexpensive, rapid and non-invasive procedures that can assist the clinician in distinguishing OPMDs from healthy mucosa but they are not able to distinguish benign and malignant lesions.

## 1. Introduction

Lip and oral cavity cancer is the 16th most common neoplasm in the world with 377,713 new diagnoses and 177,757 deaths estimated in 2020 [1,2]. The most frequent malignant neoplasm in the mouth is the oral squamous cell carcinoma (OSCC); it accounts for about 90–95% of the cases, and the remaining 5–10% consist of rarer malignancies [3,4]. To date, OSCC is the seventh leading cause of death in males between 30 and 70 years old and the 14th in women of all ages; the female/male ratio is 1:2 [2]. In recent years, there have been many improvements in diagnosis and treatment, but the five-year survival of OSCC remains low at less than 50%, even if morbidity and prognosis are directly related to the stage of diagnosis [5]. OSCC may arise *de novo* or from oral potentially malignant disorders (OPMDs) [6,7]. For this reason early detection of OPMDs could improve OSCC prognosis [8].

OPMDs include several oral conditions and diseases as leukoplakia, erythroplakia, oral lichen planus (OLP) and submucosal fibrosis that have a high potential for neoplastic transformation. For example, according to Giuliani et al. [9], the risk of malignant transformation for oral lichen planus is 1.40%, and in their systematic review, 92 of 6559 patients with OLP developed oral squamous cell carcinoma, reporting a rate of neoplastic evolution of 0.20% in a year. Among OPMDs oral leukoplakia is the most frequent precancerous lesion of the oral mucosa; its global prevalence is approximately 2.60% [10]. Warnakulasuriya et al. [11] show how the rate of malignant evolution for leukoplakia varies from 0.13 to 34%. A higher grade of dysplasia, advanced age, female sex, leukoplakia larger than 200 mm^2^ and non-homogeneous clinical aspect are the main risk factors for malignant transformation [11]. Detection and diagnosis of OPMDs, however, can be complex because these disorders may be asymptomatic or mimic some benign conditions (e.g., reactive or inflammatory lesions of the oral mucosa). Biopsy with histopathological examination, therefore, is required for definitive diagnosis and it currently remains the gold standard for OPMDs and OSCC diagnosis and management [12].

Neoplastic transformation of oral lesions is related to several risk factors: smoking or chewing tobacco, alcohol abuse, bad oral hygiene, chronic trauma and human papilloma virus infections (HPV) [13,14], but the most important predictor of malignant development is the presence of epithelial dysplasia [15]. According to Sperandio et al., around 0.01% of non-dysplastic lesions and a variable percentage of dysplastic lesions (from 6% to 39%) can evolve into neoplastic lesions, and the percentage of malignant transformation increases when the degree of dysplasia grows [15]. Warnakulasuriya et al. show the association between the grade of dysplasia and the neoplastic transformation: patients with severe dysplasia have a higher risk of developing an OSCC in relation to patients with suspicious oral lesion without dysplasia [16,17]. However, there is no a distinctive clinical presentation of dysplasia, so it is not possible to visualize which parts of a suspicious lesion may contain dysplasia areas until performing a biopsy [18]. The presence of dysplasia is the most predictive test for malignant evolution but its grading is subjective and poorly reproducible; in fact, for overcoming this issue and improving reproducibility, anatomic pathologists suggest the adoption of a binary classification of oral dysplasia (low risk and high risk) [19,20].

A DNA ploidy exam has been suggested as an alternative prognostic marker of OPMDs where aneuploid lesions seem to have a higher risk of neoplastic evaluation compared with diploid cases [21,22]. According to Sperandio et al. [15], DNA ploidy and dysplasia are independent processes and chromosomal instability is not necessarily accompanied by histologically detectable dysplasia. Their combined use may provide the highest predictive value for malignant transformation [15], but more studies are necessary to improve positive and negative predictive values [22].

Biopsy is important to help clinicians in establishing the definitive diagnosis and to check the eventual malignant transformation of OPMDs [23]. Depending on the technique employed, biopsies can be classified as incisional or excisional. In the incisional biopsy, a single part of the suspicious lesion is removed, while in the excisional biopsy, all the mucosal defect is resected. Incisional biopsy is preferred with large, doubtful and multifocal lesions; an excisional biopsy is generally applicable with benign conditions such as papillomas, fibromas or granulomas [3]. In the case of an incisional biopsy, the main problem is selection of the site so that it includes the most representative part of the suspected lesion, preferably including both normal and abnormal tissue. This approach is recommended in cases of precancerous and suspected malignancy conditions or when the lesion is too large or located in complicated areas; in cases of extensive and heterogeneous lesions, many samples should be obtained and analyzed [23]. Furthermore, while it is easy to identify clearly neoplastic lesions, dysplastic lesions or early-stage tumor are not always clinically characterized as cancer, thus mimicking oral lesions [4]. For all these reasons, several adjuncts and devices capable of providing additional information during clinical examination have been developed to assist clinicians in biopsy site selection; these include toluidine blue staining, autofluorescence, narrow-band imaging, chemiluminescence, oral exfoliative cytology [24,25,26].

During carcinogenesis, tissue structures and cell metabolism change [16] for example collagen and elastine fibres are degraded, whereas cell replication and vascularization increase [25,26,27,28,29,30]. Several diagnostic adjunct tools, such as toluidine blue staining and autofluorescence, may detect some of these distortions. Toluidine blue is the most common vital staining system in dentistry. It is a cationic metachromatic dye that stains nucleic acids that generally increase in neoplastic progression as much as in inflammatory conditions. It is able to stain malignant cells due to an increased amount of nucleic acids, a faster cell division, and wider intracellular canals, which facilitate the penetration of the dye into neoplastic cells [30,31,32,33]. Autofluorescence is a process used not only in dentistry but also in the screening and management of cervix, lung and skin tumors [34,35]. This system is based on the physiological presence of endogenous fluorochromes in the oral mucosa. These molecules, such as nicotinamide adenine dinucleotide (NADH), flavin adenine dinucleotide (FAD), collagen, elastine, keratin and hemoglobin, emit fluorescence in the green spectral range if excited by light with a wavelength of 370–460 nm. In neoplastic progression there are qualitative and quantitative changes in fluorochromes concentration [36]; for example, there is a disruption of cross-linked collagen and a reduction of the ratio of FAD to NADH since normal cells are dominated by oxidative phosphorylation whereas reductive glycolysis is most active in malignant cells. For this reason, normal tissues appear as light green fluorescence, whereas neoplastic and pre-neoplastic tissues show a loss of autofluorescence so they appear as dark green/black areas [37]. The filter Glasses for Oral Cancer Curing Light Exposed (GOCCLES^®^) is a medical device (Pierrel S.p.A-Italy) approved by the Food and Drug Administration in 2015. The optical filters consist of a three-layer laminar optical structure that allows isolation of the fluorescent component emanating from the FAD (515 nm) while excluding the other components in the visible and in the ultraviolet. It is an adjunctive tool used alongside the traditional intraoral oral examination for early detection of suspicious lesions. It is not able to distinguish between benign and malignant lesions; hence, it cannot replace histological analysis for a definitive diagnosis, but it can help to delineate the margins of a suspected lesion before surgical excision and to identify early lesions that may not be detected on clinical examination with naked eye [38,39].

The objective of this study is to calculate, in a population of patients with OPMDs, the sensitivity, specificity, accuracy, positive and negative predictive value of the optical filter GOCCLES^®^ for autofluorescence and of blue toluidine staining.

## 2. Materials and Methods

The study protocol was approved by the Ethical Committee of the A. Gemelli Foundation Teaching Hospital (approval No: 0016746/21). In this retrospective study, from January to April 2021, medical records, photographs and videos of patients with oral lesions were analyzed. Patients with OPMDs for which they needed a biopsy were recruited from the Oral Medicine Department of the A. Gemelli Foundation Teaching Hospital, from January 2018 to July 2020.

Inclusion criteria: patients at least 18 years old with at least one suspicious oral mucosa lesion evaluated in white light, autofluorescence with optical filter GOCCLES^®^, toluidine blue staining and then biopsied with histopathological analysis who had signed the dedicated informed consent. All the included patients underwent the same routinary diagnostic pathway. Those patients whose data for the biopsy, toluidine blue staining and autofluorescence could not be traced were excluded, as well as patients for whom the graphic resolution of photos and/or videos did not allow identification of the study outcomes. All the examinations were performed in a single appointment by the same senior specialist in oral pathology. Data about age, sex, tobacco and alcohol abuse, systemic pathologies and drug therapies were collected.

The clinical white light examination was conducted to record the localization of lesions, make a provisional clinical diagnosis and give a dichotomous value according to presence or absence of suspicious premalignant or malignant lesions. According to their clinical appearance, the lesions were classified into three groups: lichenoid lesions (characterized by the typical reticular distribution of white lesions, with or without erosions or ulcerations), erytroplakia-leucoplakia-erytroleukoplakia (clinically red or white lesions or with white and red spots), and verrucous leukoplakia (exophytic lesions with proliferative features).

**Figure 1:**
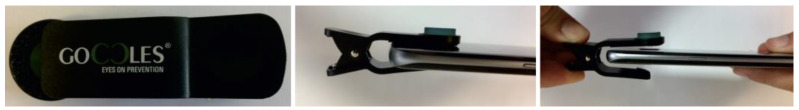
The GOCCLES^®^ clip (Glasses for Oral Cancer Curing Light Exposed Screening) medical device.

The autofluorescence examination was taken using the GOCCLES^®^ optical filter in the form of clip attached to a smartphone camera in order to take pictures and/or videos (Figure 1). During this examination, the room lights were turned off and a dental curing lamp was used as light source at a distance of about 20 cm and perpendicular to the inspected area. Lesions that showed a loss of fluorescence appeared dark green/black and were classified as positive (FVL-Fluorescence visualization loss), whereas lesions appearing light green were classified as negative (FVR-Fluorescence visualization retained).

The same lesions were, subsequently, evaluated using blue toluidine staining, according to the Mashberg technique [40] and, according to the color intensity; they were classified as Dark Royal Blue (positive) or Pale Royal Blue (negative) [41,42].

All the lesions were biopsied and the site selection was guided by irregular clinical appearance, loss of autofluorescence and positivity to toluidine blue staining. Areas that showed simultaneously all three of these aspects were preferred over sites with only two or just one of the mentioned features. For the histopathological evaluation of the biopsied lesions, the assessment of positivity was given in the presence of dysplasia and/or carcinoma. The biopsies were fixed in formalin and the histopathological analyses were conducted by the department of Pathological Anatomy of the A. Gemelli Foundation Teaching Hospital.

In cases with multiple lesions, more than one sample was taken from the same patient. For statistical purposes, each specimen was considered as a single biopsy and each lesion was evaluated independently from the others. All the above-mentioned procedures (clinical white light examinations, blue toluidine staining, autofluorescence analysis and biopsies) were performed in a single appointment by the same senior specialist (C.L.) with more than 10 years of experience in the oral medicine field. After every step of the diagnosis, pictures and short videos were acquired for documentation and, thus, were available for the retrospective analysis. The clinical charts, clinical pictures and videos were retrospectively examined by two authors (R.P. and M.T.), who were blinded on the histology results, and independently classified the lesions according to the clinical pictures before judging the results of the autofluorescence and toluidine blue staining. In cases of disagreement, a third author (A.M) was asked to review the records and multimedia files.

The sample size calculation was performed calculating the AUC of the ROC curve [43] considering an alpha of 0.05% and an 80% power, an AUC value for toluidine blue of 0.5208 [44] and a hypothetical value of 0.8 for the optical filter GOCCLES^®^, for which it would be necessary to include 31 samples. The sample was described in its clinical and demographic characteristics through descriptive statistical techniques. In particular, the quantitative variables were represented through the following measures: minimum, maximum, range, mean and standard deviation. Area under the curve (AUC), sensitivity, specificity, and positive and negative predictive values were calculated for toluidine blue and GOCCLES^®^ optical filter and compared with histological examination. The qualitative variables were described with tables of absolute frequencies and percentages. The normality of the continuous variables was verified with the Kolmogorov–Smirnov test, the type of distribution with the Skewness Kurtosis test, and the differences between injury types using ANOVA or Kruskal–Wallis. The dispersion of the average values was indicated with SD and IQR. The statistical analysis was performed with specific software (STATA 15; STATA Corp LLC, College Station, Texas, USA).

## 3. Results

In this study, a total of 25 patients including 13 females (52%) and 12 males (48%) with an average age of 67.4 years (SD: 15.75) and an age range of 35–88 years, were recruited. From the abovementioned patients 42 areas were biopsied to offset eventual drop-outs. Only forty-one biopsies were analyzed since in one case, multi-media were not adequate to make a proper clinical evaluation. Three patients were current smokers, seven were ex-smokers and fifteen were non-smokers; among the three smokers only one was also a regular alcohol user; among the remaining 24 patients there were no drinkers. Table 1 shows the demographic characteristics of the study sample.

Biopsies were performed in all the 25 patients after a clinical examination in white light, an autofluorescence examination with the GOCCLES^®^ filter and a blue toluidine staining. In ten cases, for establishing a more accurate histological diagnosis, more than one biopsy per patient was performed, but each specimen was considered as a distinct biopsy. Data concerning the location of the samples, clinical appearance and provisional clinical diagnosis are summarized in Table 2, Table 3 and Table 4.

### 3.1. GOCCLES^®^

Eighteen lesions showed no fluorescence loss and were considered negative (FVR); fourteen of these eighteen samples revealed a benign histology and, therefore, were classified as true negatives. However, four lesions were rated as false-negative, being diffuse proliferative lesions that showed, after the histological examination, moderate dysplasia in one case and three verrucous proliferative carcinomas in the remaining ones. In 23 lesions GOCCLES^®^ revealed loss of autofluorescence, so these were classified as positive (FVL), but only eight of this group showed positive histology; therefore, they were considered as true positive. Clinically, such cases were erythroplakia with a suspected diagnosis of carcinoma; histologically, four of them were severe, three moderate and one mild dysplasia. Fifteen samples with loss of autofluorescence (FVL) and benign histology were considered false positives, and clinically, two of them had proliferative verrucous features with suspected carcinoma, six were lichenoid lesions with suspected OLP, seven were erythroplakias including three suspected OLP, three were erythroplakias and one was suspected as an ulcer.

The sensitivity and specificity for the autofluorescence evaluation with the GOCCLES^®^ filter for identifying dysplasia or carcinoma were 66% and 48%, respectively. The positive and negative predictive values were 34% and 77%, respectively; the accuracy was 53%.

### 3.2. Blue Toluidine

All the 41 lesions were also subjected to toluidine blue staining. A total of 20 lesions were classified as Dark Royal Blue according to the dye intensity; the remaining 21 lesions showed no coloration and therefore were classified as Pale Royal Blue. Eleven of the twenty Dark Royal Blue lesions showed a positive histology; hence, they were classified as true positive. Among these, three lesions had the clinical feature of proliferative verrucous leukoplakia, and revealed moderate dysplasia; the other two received a histological diagnosis of verrucous proliferative carcinoma. The other eight lesions appeared clinically as erythroplakia and three were moderate dysplasia, four severe dysplasia and one mild dysplasia. The remaining nine Dark Royal Blue lesions without dysplasia and/or carcinoma were classified as false positives: two of them had the clinical feature of lichenoid lesions, one of leukoplakia and four of erythroplakia. Only one lesion, with a clinical proliferative appearance and a histology of verrucous proliferative carcinomas, was rated as a false negative. Twenty Pale Royal Blue lesions showed a negative histology and were classified as true negatives.

**Figure 2:**
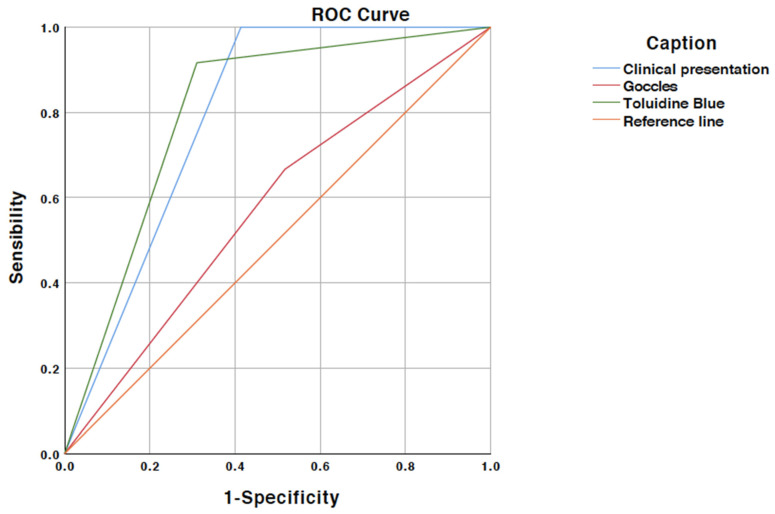
Roc Curve.

The sensitivity and specificity for toluidine blue staining were 91% and 68%, respectively. The positive and negative predictive values were 55% and 95%, respectively; the accuracy was 75%. The data for these results are summarized in Table 5 and Table 6. The ROC space analysis is shown in Figure 2.

## 4. Discussion

This study evaluated the use of the optical filter GOCCLES^®^ for autofluorescence and of blue toluidine staining as adjunctive diagnostic methods to identify dysplastic areas and/or epithelial neoplasia in patients having suspected oral lesions.

**Figure 3:**
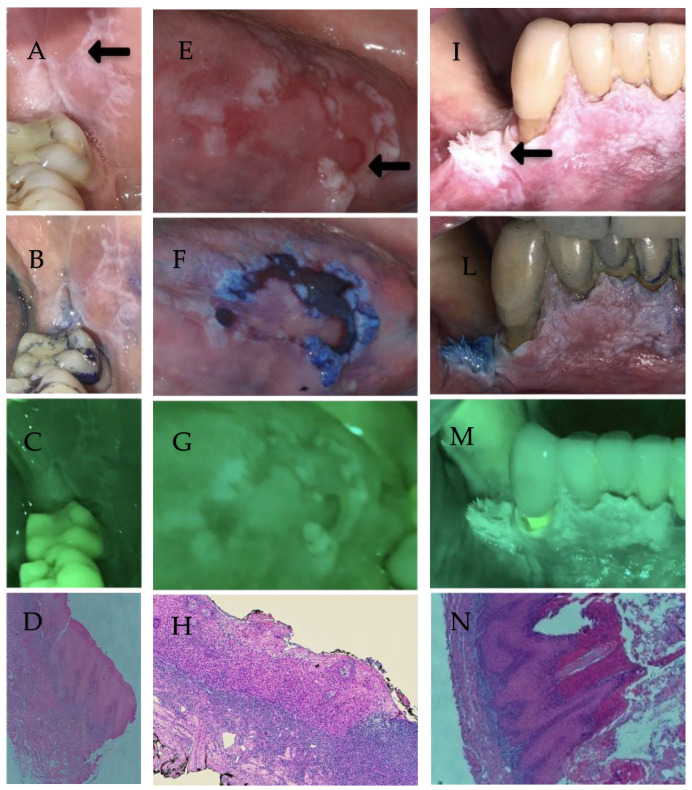
Clinical appearance, blue toluidine staining and autofluorescence analysis of three different lesions. Case 1 shows a lichenoid lesion in white light (**A**) considered non-suspicious at clinical examination, which was negative by blue toluidine staining (**B**) and autofluorescence visualization (**C**). The histopathologic analysis shows acanthosis, hyperparacheratosis, infiltrated lichenoid lymphocyte with vacuolization of the basal layer (**D**). Case 2 shows a suspicious red lesion (**E**), that was positive by blue toluidine staining (**F**) and autofluorescence visualization (**G**). Biopsy (**H**) revealed a condition of moderate dysplasia with areas of severe dysplasia. Case 3 shows a proliferative suspicious lesion (**I**) that was negative by blue toluidine staining (**L**) and autofluorescence visualizaion (**M**). The histopathologic diagnosis was a verrucous carcinoma (**N**).

The sensitivity and specificity of GOCCLES^®^ were 66% and 48%, respectively, and the filter proved to be useful in identifying dysplastic areas in red lesions. in fact, it detected the only case of mild dysplasia, 75% of the moderate dysplasia with one false negative due to the proliferative feature of the lesion, and 100% of the severe dysplasia. Autofluorescence was unable to identify any case of verrucous carcinomas, recording four false negative results, all of them in proliferative lesions. The risk of false negatives using autofluorescence is described in literature for lesions with hyperkeratosis since the thick layer of keratin, which is an important endogenous fluorophore, can cause an increase of fluorescence that may hide the dysplastic and/or neoplastic areas, causing what is referred as the *umbrella effect* [45,46] (Figure 3). This problem is already known in the diagnostic and therapy field; for example, the photodynamic therapy (PDT), another system based on the interaction of a light source and the application of local or systemic photosensitizer, is less efficient in detecting proliferative lesions because the presence of epithelial hyperkeratosis hinders the penetration of this substance. PDT, as the autofluorescence system, uses the different concentrations of fluorophores in premalignant and malignant areas, due to an altered metabolism in tumor cells, for identifying suspicious sites. This procedure is also proposed as an alternative treatment for OPMD because the interaction between a specific wavelength of a light source and the photosensitizer produces singlet oxygen and free radicals, which cause oxidative damage and cell death [47,48].

Otherwise, in the case of lesions suspected to be ulcers or OLP, due to the inflammatory nature of diseases, there is a higher risk of false positives; six of the nine lichenoid lesions with suspected OLP and the only case of a lesion suspected to be an ulcer showed loss of fluorescence even without dysplasia, resulting in false positives. In the literature, to overcome this problem, it is proposed to eliminate all the possible local irritant factors and then re-evaluate the same lesions after 2 weeks in order to exclude traumatic or acute inflammatory lesions which would cause some false positives [49]; moreover, in the literature, several articles suggest to accurately evaluate the localization of the lesions because there are some areas, such as the vermilion or the dorsum of tongue, with a high risk of false positives due to a greater physiological vascularity [41,50]. The main reason for the low sensitivity compared with the literature is the inclusion of lesions of different natures and clinical features (i.e., lichenoid lesions, verrucous lesions, leukoplakia etc.), since GOCCLES^®^ is, for example, less powerful at predicting the progress of the disease in white lesions than in red lesions. For example, Koch et al. [45] show a sensitivity of 96% in identifying dysplasia and carcinoma, but the same authors admit that the high values are related to clearly malignant lesions, and they also claim that sensitivity decreases as the layer of keratosis increases. For example, the study of Paderni et al., in which 66% of the samples were white lesions, described a sensitivity of 75%, which is relatively low, for VELscope^®^ (one of the most commonly used devices for autofluorescence) [49]. According to Canjau et al. [50], autofluorescence cannot replace histological examination for the diagnosis of suspicious oral lesions but it is an economic and non-invasive procedure capable of increasing the sensitivity of conventional clinical examination. For Amirchagmachi et al., autofluorescence devices are useful as adjunct tools but they are unable to discriminate benign lesions from malignant ones [51]. Cicciù et al. [52,53], however, suggest using these tools for identifying the most appropriate area to take a biopsy in cases of suspected lesions. Moreover, the results of the current study are not completely consistent with the results of Moro et al. in two different studies performed with the analogous GOCCLES^®^ optical filter in the form of glasses [38,54] since the objective of the authors was to evaluate this filter as a screening test for early detection of oral carcinoma in a population at high risk of developing oral cancer.

Literature data about blue toluidine staining show high sensitivity and low specificity (although higher than autofluorescence). A review published in 2012 reported a sensitivity between 93.5% and 97.8%, and a specificity between 73.3% and 92% [55]. The present study described a sensitivity of 91% and a specificity of 68% in accordance with literature data, and the effectiveness in identifying dysplastic and/or neoplastic areas was greater for red lesions; moreover, lesions with suspected OLP showed an increased risk of false positives, similarly to autofluorescence. Martin et al. [56] describe a sensitivity of 100% for carcinomas, but they found a sensitivity of 75% for dysplastic lesions; similar values are described by Warnakulasuriya et al. [57] who found sensitivity and specificity of 74% and 66%, respectively. Onofre et al. [58] report that toluidine blue is able to identify only 50% of dysplastic lesions but 100% of lesions with carcinoma, while 35% of benign lesions were positive, thus showing the difficulty in distinguishing benign from malignant lesions. However, in contrast to the GOCCLES^®^ optical filter, toluidine blue was also able to identify verrucous carcinomas, except in one case. It must be wondered if the increased intensity of the dye on verrucous-proliferative lesions is actually associated with the ability of toluidine blue to identify areas with higher replication of nucleic acids or if it is just a consequence of mechanical retention caused by the porous surface of the lesion.

It is important to note that it requires some practice for users of the GOCCLES^®^ optical filter to develop the ability to take appropriate pictures, isolate the field to avoid shadow cones, and interpret images properly. The procedure for toluidine blue staining is much simpler, but inter-operator variability related to the subjective judgment is ever present since the intensity of blue is not associated with an objective scale, despite the Dark Royal Blue and Pale Royal Blue distinction. A color quantitative analysis was proposed in the early detection of esophagus carcinomas to overcome the subjective interpretation of results. Guneri et al. performed a computer analysis that defined the association between the blue scale and the malignancy of lesions [59]. Similarly, to overcome the bias of individual interpretation of autofluorescence, Huang et al. [60] proposed a quantitative analysis of the results of the VELscope^®^ that shows promising results with a specificity of 92% and a sensitivity of 97% in distinguishing neoplastic mucosa from normal ones, although the ability of distinguishing abnormal tissue from normal tissue in cases of early oral cancerous lesions is more complex because the clinical aspects are less significant.

The present study has some limitations, which need to be underlined. First, it includes lesions with different clinical features and nature; this aspect could explain why results of the autofluorescence are different compared with those in the literature. Second, this is a retrospective study on a relatively small cohort of patients; bigger prospective multi-center studies should be performed to confirm the data obtained. Another limitation is the subjective considerations about the results of autofluorescence and blue staining toluidine since there is not an objective scale for classifying the features of the lesions.

## 5. Conclusions

In conclusion, the study shows that autofluorescence and toluidine blue staining are simple, inexpensive, rapid and non-invasive procedures, but the interpretation of the result is closely related to operator experience and knowledge. These methods are able to identify suspected lesions but they are not able to differentiate between benign and malignant lesions; for this reason they must always be compared with histopathological examination. These procedures, however, can help to maximize diagnostic performance, extend the limits of the oral clinical exam and encourage clinicians to perform more accurate examination of the oral cavity, so as to promote the culture of early detection of oral carcinoma.

Clinical training, especially in oral pathology, has particular importance because lesions of a different nature can have similar clinical features, and at the same time, a single disease can show several clinical aspects.

## Figures and Tables

**Table 1:** Demographic information of patients examined (*n* = 25).

Characteristic	Male (*n* = 12)	Female (*n* = 13)
Age mean, y	56, 75 (SD 14, 97)	75, 69 (SD 9, 58)
Smokers, *n*	2	1
Ex-smokers, *n*	4	3
Non-smokers, *n*	6	9
Alcohol consumer, *n*	1	0
Smoker & alcohol, *n*	1	0

**Table 2:** Location of biopsied lesions (*n* = 42).

Location	*n*
Tongue (Dorsum)	1
Cheek	16
Floor of the mouth	2
Gingiva	5
Palate	7
Lip	1
Retromolar region	2
Tongue (Inferior Surface)	8

**Table 3:** Clinical appearance of biopsied lesions (*n* = 42).

Clinical Features	*n*
LVP	11
L	9
ER	21

Abbreviations: LVP, verrucous proliferative leukoplakia; L, leukoplakia; ER, erythroplakia.

**Table 4:** Provisional clinical diagnosis (*n* = 42).

Clinical Diagnosis	*n*
Carcinoma	17
Oral Lichen Planus	13
Erythroleukoplakia	1
Leukoplakia	5
Ulcera	1
Erythroplakia	4

**Table 5:** Correlation of GOCCLES^®^ examination and Toluidine Blue staining with histopathological findings (*n =* 41 lesions).

	TP, *n*	TN, *n*	FP, *n*	FN, *n*	Sensitivity	Specificity	PPV	NPV	Accuracy
GOCCLES^®^	8	14	15	4	66%	48%	0.34	0.77	0.53
Toluidine Blue staining	11	20	9	1	91%	68%	0.55	0.95	0.75

Abbreviations: TP, true positive; TN, true negative; FP, false positive; FN, false negative; PPV, predictive positive value; NPV, predictive negative value.

**Table 6:** Histological results (*n =* 41).

Histology	*n*
Mild Dysplasia	1
Moderate Dysplasia	4
Severe Dysplasia	4
Verrucous Carcinoma	3
Benign histology	29

## Data Availability

Data are available upon request to the authors.
